# First international summit on fibrosis in intestinal inflammation: mechanisms and biological therapies

**DOI:** 10.1186/1755-1536-3-22

**Published:** 2010-11-11

**Authors:** Florian Rieder, Claudio Fiocchi

**Affiliations:** 1 Department of Gastroenterology & Hepatology, Digestive Disease Institute, The Cleveland Clinic, Cleveland, OH, USA; 2 Department of Pathobiology, Lerner Research Institute, The Cleveland Clinic, Cleveland, OH, USA

## Abstract

The first meeting dedicated to intestinal fibrosis, entitled *The First International Summit on Fibrosis in Intestinal Inflammation: Mechanisms and Biological Therapies*, was held in Cleveland, Ohio, USA, on 28-29 September 2010. Intestinal fibrosis is a complication of inflammatory conditions affecting the small and large bowel and often results in serious clinical consequences. Despite its clinical importance, the study of inflammation-driven intestinal fibrosis has received very limited attention. This explains why so little is known about its pathophysiology and the lack of significant therapeutic advances, in contrast with the recent success achieved in controlling gut inflammation with biological agents. The meeting covered most aspects directly relevant to intestinal fibrosis, including gut inflammation; cellular and molecular mechanisms of intestinal fibrogenesis; new clinical, diagnostic and prognostic tests and novel therapeutic approaches.

## Introduction

Intestinal fibrosis is common in chronic intestinal inflammation as typically observed in both forms of inflammatory bowel disease (IBD), Crohn's disease and ulcerative colitis. Fibrosis is a particularly frequent and serious complication in Crohn's disease, where it causes clinically symptomatic narrowing of the lumen that often requires surgical intervention. Unlike fibrosis involving other organs, such as the liver and the lung, where knowledge of the underlying pathogenic mechanisms is more advanced, the study of intestinal fibrosis has received very limited attention so far. This largely explains the lack of understanding of its cause and mechanisms and the exceedingly restricted and largely ineffective therapeutic options available for medical therapy. To improve this situation, generate new knowledge and promote advances in this important clinical area, *The First International Summit on Fibrosis in Intestinal Inflammation: Mechanisms and Biological Therapies *was held at the Cleveland Clinic, Cleveland, Ohio, USA, on 28-29 September 2010 (Additional file 1). The summit was attended by approximately 90 participants from the USA, Europe and Asia, including speakers, young investigators and a broad audience of basic investigators, clinicians, gastroenterologists, surgeons and health-related personnel.

## Summary and Discussion

The summit unfolded over a 2-day period and was primarily focused on fibrosis of the small and large intestine, with one session dedicated to liver fibrosis and one session devoted to endoscopic approaches to gastrointestinal strictures and chronic pancreatitis. In the opening remarks, Dr. Victor Fazio (Digestive Disease Institute and Colorectal Surgery, Cleveland Clinic, Cleveland, OH, USA) provided an overview of intestinal fibrosis from a clinical and surgical perspective, underscoring the magnitude of the clinical problem, the evolution of surgical techniques (strictureplasty) to relieve stenotic lesions in Crohn's disease, and the urgent need to gain a better understanding of the pathophysiology of stricture formation.

Session I was dedicated to the mechanisms and therapy of inflammation. Dr. Claudio Fiocchi (Department of Gastroenterology and Hepatology and Department of Pathobiology, Cleveland Clinic) stressed the close interrelationship between chronic inflammation and intestinal fibrosis and reviewed classical and novel cellular and molecular mechanisms leading to activation of myofibroblasts, the primary culprits responsible for excessive deposition of collagen and other extracellular matrix (ECM) proteins in fibrotic conditions. Dr. Silvio Danese (Division of Gastroenterology, Istituto Clinico Humanitas, Milan, Italy) discussed the increasingly appreciated notion that intestinal inflammation is not the exclusive result of immune activation, but instead the outcome of complex interactions between immune and nonimmune cells. In addition, Dr. Danese pointed out that an exaggerated emphasis on the role of the mucosal immune system as the arbitrator of IBD can be detrimental to a comprehensive and more realistic view of IBD pathogenesis and an explanation to the lack of attention given to other cell types, particularly the mesenchymal cells responsible for intestinal fibrosis. Dr. Georg Wick (Laboratory of Autoimmunity, Innsbruck Medical University, Innsbruck, Austria) reviewed classical concepts of immunology in the context of clinically relevant fibrotic diseases, using as an example the fibrosis that develops around silicone breast implants, with the local and systemic consequences of this unique fibrotic response. Similarities and differences with intestinal fibrosis were discussed. Dr. Leonard Calabrese (Department of Rheumatic and Immunologic Diseases, Cleveland Clinic) closed Session I with a broad overview of the development, current use and future applications of biological agents in the management of autoimmune and chronic inflammation diseases, as well as the effect of such agents on the fibrotic response that is often associated with many of those conditions.

Session II was devoted to specific mechanisms of fibrogenesis. Dr. Thomas Wynn (Immunopathogenesis Section, National Institute of Allergy and Immunological Diseases, National Institutes of Health, Bethesda, Maryland, USA) initiated his talk by reminding the audience that almost one-half of all natural deaths in the developed world are attributable to fibroproliferative disorders. He then reviewed recent evidence demonstrating that interleukin (IL)-13 signaling through the IL-13Rα2 is involved in induction of TGF-β1 production and fibrosis and discussed the distinct and overlapping roles for IL-13 and IL-17 in murine *S. mansoni*- and bleomycin-induced liver and pulmonary fibrosis models, respectively. The central role of tumor growth factor (TGF)-β in fibrosis was briefly reviewed by Dr. Rik Derynck (University of California San Francisco, San Francisco, California, USA), who then elaborated in greater detail on the role of TGF-β in the intriguing but controversial topic of epithelial-to-mesenchymal transition (EMT). EMT and related processes, such as endothelial-to-mesenchymal transition (EndoMT), represent examples of processes in which nonmesenchymal cell types are coerced into *de novo *collagen-producing cells under immune and inflammatory pressure. This is conceptually novel and challenges the conventional view that only mesenchymal cells (fibroblasts, myofibroblasts, muscle cells and stellate cells) produce excessive ECM during a fibrogenic response. Dr. Subrata Ghosh (University of Calgary, Calgary, Alberta, Canada) approached the challenging topic of whether TNF-α is a pro- or antifibrotic agent. Some early reports claimed that Crohn's disease patients given the anti-tumor necrosis factor (TNF)-α monoclonal antibody infliximab experienced worsening of preexisting strictures. This led to the highly debated issue of whether infliximab or other anti-TNF-α agents might be contraindicated in Crohn's disease patients carrying important stenotic lesions. After a detailed review of the literature, Dr. Ghosh concluded that current data do not support the contention that intestinal stenosis, stricture or obstruction are precipitated by such agents in the majority of Crohn's disease patients. Session II was concluded by Dr. Donald Powell (The University of Texas Medical Branch, Galveston, Texas, USA), who presented in depth information on the intestinal myofibroblast; its multiple sources (e.g., bone marrow stem cells, circulating fibrocytes and epithelial and endothelial cells); its diverse biological functions in gut inflammation, repair and fibrosis; and the possibility that intestinal myofibroblasts might be a target for stem cell therapy.

The first day ended with Session III, which was dedicated to presentations by young investigators, either junior faculty members or fellows in training, who came to the summit from the USA, Germany, The Netherlands, Italy, Spain and Belgium. They presented ongoing research on a variety of clinical and basic science topics related to intestinal fibrosis. These included new imaging modalities to detect intestinal fibrosis in Crohn's disease patients and murine models of IBD, identification of transcription factors and cytokines involved in the *S. typhimurium *model of inflammation-driven fibrosis, EndoMT in human and experimental IBD, the imbalance of matrix metalloproteinases (MMPs) and tissue inhibitor of metalloproteinases (TIMPs), and the role of IL-10, IL-12/23(p40) and IL-13Rα2 in murine schistosomiasis-associated liver fibrosis, the molecular mechanisms of acute chronic inflammation and fibrosis in sepsis, the suppressive role of the adipokine C1q/TNF-related protein-3 (CTRP-3) on inflammatory and fibrotic responses of primary human colonic fibroblasts and the role of eosinophils in murine intestinal inflammation and remodeling.

The second day of the summit started with Session IV, which centered on mechanisms of fibrogenesis in the gastrointestinal tract. Opening this session, Dr. Thomas Wight (Benaroya Research Institute and the University of Washington, Seattle, Washington, USA) approached the question whether the ECM is an active or passive player in fibrosis. He presented evidence for the close reciprocal relationship existing between cells and the surrounding ECM, as well as its broad impact on cell adhesion, proliferation, migration and survival. He went on by reminding the audience that ECM changes leading to fibrosis are common in several diseases and exemplified this by enumerating the various types of ECM proteins found in postmyocardial infarction scars and the differential binding of T cells and monocytes to different ECM components. The topic of ECM continued with a presentation by Dr. Carol de la Motte (Department of Pathobiology, Cleveland Clinic) on the role of hyaluronan in inflammation in general and intestinal inflammation in particular. Hyaluronan is rapidly produced upon tissue injury to provide a temporary matrix to maintain structural integrity, but upon its fragmentation size-specific fragments of hyaluronan can be proinflammatory, and clearance of hyaluronan is required for resolution of inflammation. The direct relationship between hyaluronan and intestinal fibrosis remains a topic of further exploration. Human and animal models of fibrogenesis in gut inflammation were discussed by Dr. P. Kay Lund (University of North Carolina at Chapel Hill, Chapel Hill, North Carolina, USA). While fibrosis occurs in various intestinal diseases, such as Crohn's disease, ulcerative colitis, collagenous colitis and radiation injury, human data are limited to descriptive studies describing the presence of diverse mesenchymal cell types based on the expression of α-smooth muscle actin, vimentin, desmin or c-kit. More precise functional information can be derived from animal models of experimental IBD and fibrosis, and recently this field has received deserved attention with the use of modified models of colitis (trinitrobenzene sulfonic acid-, dextran sodium sulfate- and *S. typhimurium*-induced and IL-10 deficiency) and ileocolitis (peptidoglycan-polysaccharide [PGPS] -induced, senescence-accelerated mouse P/Yit [SAMP/Yit], TNF-αΔ^ARE ^and ileocolic resection in IL-10-deficient mice). Each model has advantages and disadvantages, but these models do offer the distinct advantage of allowing the study of gut fibrosis right after the triggering insult. Session IV was closed by the presentation of Dr. Miquel Sans (Hospital Clínic/IDIBAPS, Barcelona, Spain), who introduced the concept of an imbalance between intestinal fibroblast proliferation and apoptosis in IBD, which could contribute to fibrogenesis in Crohn's disease and ulcerative colitis. In addition, he presented evidence that the vitamin E derivates tocotrienols inhibit human intestinal fibroblast proliferation and induce their apoptosis, possibly though caspase 3- and 8-mediated mechanisms. These effects could make tocotrienols suitable to prevent or suppress intestinal fibrosis.

Clinical and therapeutic aspects of intestinal fibrosis were discussed in Session V. Dr. Joseph Willis (Case Western Reserve University, Cleveland, Ohio, USA) reviewed the key pathological features of intestinal fibrosis in IBD and other conditions, underscoring that fibrosis is present not only in Crohn's disease but also ulcerative colitis, although it is usually limited to the submucosal layer in the latter condition. New imaging modalities to detect and localize fibrosis in the gastrointestinal tract were introduced by Dr. Ellen Zimmermann (University of Michigan, Ann Arbor, Michigan, USA). She went over what computed tomographic (CT) enterography, magnetic resonance (MR) enterography, magnetization transfer magnetic resonance imaging and ultrasound elastography can offer in the evaluation of Crohn's disease-affected bowel, pointing to the still experimental nature of the techniques currently used. The need and potential value of developing markers for risk and outcomes in intestinal fibrosis were discussed by Dr. Florian Rieder (Department of Gastroenterology and Hepatology and Department of Pathobiology, Cleveland Clinic). Antibacterial antibodies, ECM proteins and growth factors have been proposed as markers or predictors of fibrostenotic Crohn's disease. While they are promising tools, lack of specificity for fibrosis, fluctuation over time and lack of prospective studies presently limit their value and practical clinical applicability. Dr. Shelia Violette (Stromedix, Inc., Cambridge, Massachusetts, USA) presented information on the development of a clinical biomarker strategy for fibrotic diseases. The integrin αvβ6 is highly upregulated in injured epithelial surfaces and leads to TGF-β activation and, as such, represents an important mediator of fibrosis in organs such as the liver, kidney and lung. As a result, αvβ6 could potentially serve as a marker of fibrosis but at the same time a target whose blockade could inhibit TGF-β activation and relieve fibrosis. Closing this session, Dr. Jane Onken (Duke University Medical Center, Durham, North Carolina, USA) discussed stem cell therapy for fibrosis by first enumerating properties that may make them suitable for therapy, such as regulation of the immune system, prevention of scar formation, formation of new vessels, homing to sites of injury and induction of tissue regeneration. Clinical trials with stem cells are under way in a variety of conditions, including IBD. An open-label phase II trial of stem cell therapy in Crohn's disease showed a decrease of clinical activity indices, some clinical responses and improvement in quality of life. However, important challenges remain to be addressed before a phase II trial can be undertaken, including optimal dose, durability of response, evidence of mucosal healing, biomarker availability and long-term safety.

Session VI was dedicated to liver fibrosis, an area far more developed than intestinal fibrosis. Dr. Takao Sakai (Department of Biomedical Engineering, Cleveland Clinic) reviewed the animal models currently available for study and modulation of experimental liver fibrosis, with particular emphasis on the role of ECM proteins. Dr. Massimo Pinzani (University of Florence School of Medicine, Florence, Italy) gave a state-of-the-art overview of current knowledge of the biology of hepatic stellate cells and highlighted their role in chronic hepatitis C viral infection. The serious and present-day issue of widespread obesity and its implications for insulin resistance and liver fibrosis were examined by Dr. Laura Nagy (Department of Pathobiology, Cleveland Clinic). She emphasized the worldwide increasing prevalence of nonalcoholic liver disease, obesity and fatty liver, as well as the evolution from fatty liver to steatohepatitis to cirrhosis. She portrayed adipose tissue as a proinflammatory system playing a major role in the pathogenesis of hepatic fibrosis. Session VI continued with Dr. Jonathan Fallowfield (University of Edinburgh, Edinburgh, UK), who discussed therapeutic targets in liver fibrosis. Liver cirrhosis is on the rise, and current antifibrotic approaches should ideally be based on the treatment of the underlying cause, but this is not always possible. No antifibrotic agents are presently licensed in the USA or Europe, but numerous agents are being experimentally tested. These include caspase inhibitors, peroxisome proliferator-activated receptor-γ (PPAR-γ) agonists such as pioglitazone, TGF-β blockers, colchicines, relaxin, adiponectin, endothelin A, angiotensin receptor blockers, cannabinoids and alterations of the MMP-TIMP balance. Many of the agents have been used in preclinical, phase I or phase II trials; few trials are in phase III or IV, but their clinically effectiveness remains to be established. Last, Dr. Arthur McCullough (Department of Gastroenterology and Hepatology, Cleveland Clinic) reviewed current indications of biological agents in nonalcoholic fatty liver disease, a controversial area with limited supporting evidence.

The summit concluded with Session VII devoted to endoscopic or experimental approaches to management of gastrointestinal strictures and chronic pancreatitis. Although mechanical solutions do not provide definitive solutions to biological problems, dilation of strictures, as performed in esophageal lesions, biliary tree lesions and IBD do provide significant clinical relief, as pointed out by Dr. John Vargo and Dr. Bo Shen (both from the Department of Gastroenterology and Hepatology, Cleveland Clinic) and are well warranted. Finally, Dr. Tyler Stevens (Department of Gastroenterology and Hepatology, Cleveland Clinic) reviewed therapeutic options for the management of fibrosis in chronic pancreatitis. Various agents and procedures have been proposed for this problematic clinical condition, ranging from surgical or endoscopic interventions to immune modulators, antioxidants, angiotensin receptor blockers, PPAR-γ ligands and herbal compounds.

## Competing interests

The authors declare that they have no competing interests.

## Authors' contributions

Both authors contributed equally to the conception and writing of the manuscript.

## Supplementary Material

Additional file 1 **Meeting announcement flyer**. A single-page color flyer announcing the First International Summit on Fibrosis in Intestinal Inflammation: Mechanisms and Biological Therapies, its location, time of year and contact information. Click here for file

