# Supplementary material for: First international summit on fibrosis in intestinal inflammation: mechanisms and biological therapies
Source: Fibrogenesis Tissue Repair. 2010 Nov 11;3:22. doi: 10.1186/1755-1536-3-22 (PMC2989302; doi:10.1186/1755-1536-3-22)

Cleveland Clinic
Digestive Disease Institute

Digestive Disease Institute Week

1st
International Summit on Fibrosis in Intestinal Inflammation: Mechanisms and Biological Therapies

September 28 – 29, 2010

InterContinental Hotel & Bank of America Conference Center | Cleveland, OH

The **1st International Summit on Fibrosis in Intestinal Inflammation: Mechanisms and Biological Therapies** will be held from September 28 to 29, 2010, in Cleveland, Ohio. The Summit will gather a distinguished panel of national and international experts in basic, translational and therapeutic aspects of inflammation and fibrosis in the gastrointestinal tract.

The Summit is part of a new comprehensive educational initiative entitled Cleveland Clinic Bi-Annual Digestive Disease Institute Week, and will be held at the Intercontinental Hotel, located on the main campus of the Cleveland Clinic.

The Summit will offer several fully paid travel scholarships to young investigators interested in intestinal fibrosis. Trainees, post-doctoral fellows and young faculty members interested in this field are strongly encouraged to apply so that they can present their work and attend the Summit. They can do so by contacting **clevelandclinicmeded@ccf.org** or checking the Summit web site **www.ccfcme.org/digestive10**, which will be available on May 27, 2010.

The Organizing Committee is looking forward to your participation to this unique and first of its kind meeting.

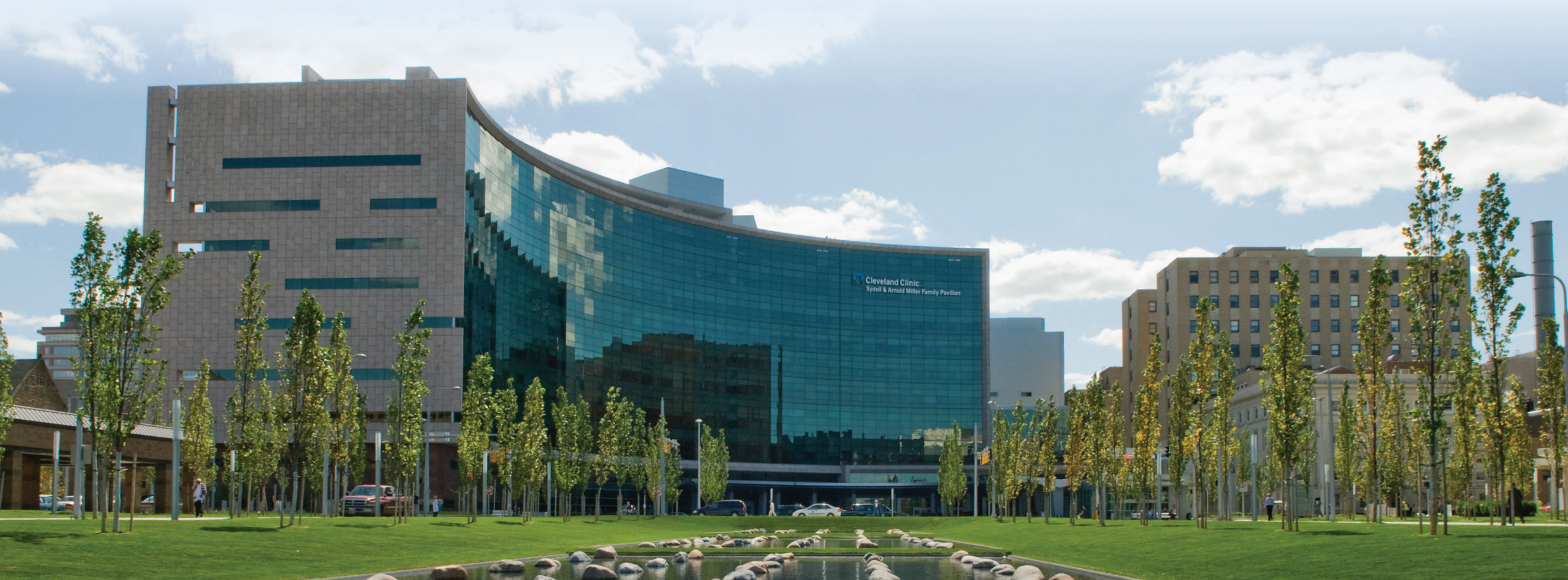

Supplement: Additional file 1 — Meeting announcement flyer. A single-page color flyer announcing the First International Summit on Fibrosis in Intestinal Inflammation: Mechanisms and Biological Therapies, its location, time of year and contact information. [file 1755-1536-3-22-S1.PDF]
